# Diaphragmatic ultrasound and patent ductus arteriosus in the newborn: A retrospective case series

**DOI:** 10.3389/fped.2023.1123939

**Published:** 2023-03-14

**Authors:** Theodore Dassios, Fahad M. S. Arattu Thodika, Mahesh Nanjundappa, Emma Williams, Aaron J. Bell, Anne Greenough

**Affiliations:** ^1^ Neonatal Intensive Care Centre, King's College Hospital NHS Foundation Trust, London, United Kingdom; ^2^ Women and Children's Health, School of Life Course Sciences, Faculty of Life Sciences and Medicine, King's College London, London, United Kingdom; ^3^ Paediatric Cardiology, Guy's and St Thomas’ Hospitals NHS Trust, London, United Kingdom

**Keywords:** diaphragmatic ultrasound, mean inspiratory velocity, prematurity, patent ductal arteriosus, infant

## Abstract

**Background:**

Patent ductus arteriosus (PDA) and diaphragmatic dysfunction are frequently seen in newborn infants but their relationship remains unknown. We aimed to use point of care ultrasound to compare diaphragmatic kinetics in infants with a PDA compared to in those without a PDA.

**Methods:**

M-mode ultrasonography was used to measure the mean inspiratory velocity (*V*_I_) in newborn infants with and without a haemodynamically significant PDA admitted in the Neonatal Unit at King's College Hospital during a three month period.

**Results:**

Seventeen diaphragmatic ultrasound studies were reviewed from 14 infants with a median (IQR) gestational age of 26.1 (25.8–30.6) weeks, birth weight of 780 (660–1385) gr at a postnatal age of 18 (14–34) days. Eight scans had evidence of a PDA. The median (IQR) *V_I_* was significantly lower in scans with a PDA [1.01 (0.78–1.86) cm/s] compared to the ones without a PDA [3.21 (2.80–3.59) cm/s, *p* < 0.001]. The median (IQR) gestational age was lower in infants with a PDA [25.8 (25.6–27.3) weeks] compared to infants without a PDA [29.0 (26.1–35.1) weeks, *p* = 0.007]. Using multivariable linear regression analysis the *V_I_* was independently associated with a PDA (adjusted *p* < 0.001) but not with the gestational age (adjusted *p* = 0.659).

**Conclusions:**

Patent ductus arteriosus was associated with a lower mean inspiratory velocity in neonates and this effect was independent of gestational age.

## Introduction

Point of care ultrasound is widely accepted in neonatal intensive care as an accessible diagnostic tool, used to diagnose lung pathology and other conditions (1). The diaphragm is the main muscle of respiration and can be assessed ultrasonographically. Measured parameters include the diaphragmatic thickness and diaphragmatic kinetics by time motion display (M-mode) ultrasonography, during which the operator can record the displacement of the diaphragmatic segments and velocity of motion (2, 3). The median diaphragmatic thickness has been reported to be higher in term compared to preterm infants highlighting increasing skeletal muscle mass (4). Hypercapnia and induced muscle fatigue by phrenic nerve pacing in piglets was associated with a significant decrease in the inspiratory velocity of the diaphragm (5).

The diaphragm undertakes the work of breathing and in the newborn, especially those born prematurely, is prone to dysfunction due to lower muscle mass, flattened shape and a low content of fatigue-resistant muscle fibres (3). In some infants impaired diaphragmatic function could clinically manifest as an inability to wean successfully from assisted ventilation (3).

Patent ductus arteriosus (PDA) is often encountered in neonatal intensive care and is a common complication of prematurity, occurring in approximately 20%–50% of neonates born before 32 weeks of gestation (6). Although PDA is considered a comorbidity that would negatively affect successful weaning from invasive ventilation (7), the relationship of PDA with diaphragmatic function has not been previously described in the neonatal population. A negative effect, however, of a significant PDA on diaphragmatic function is possible, as the pathophysiology of PDA would include some element of congestive heart failure, and numerous studies in adults have described diaphragmatic dysfunction in patients with left heart failure due to numerous distinct mechanisms (8).

We hypothesized that infants with PDA would have a different pattern of diaphragmatic kinetics assessed by diaphragmatic ultrasound compared to infants without PDA. Our aim was to test this hypothesis.

## Materials and methods

### Subjects

The echocardiograms of infants that were admitted to the neonatal unit at King's College Hospital NHS Foundation Trust, London, UK between 1 April 2022 and 30 June 2022 and underwent an echocardiographic assessment for clinical reasons were retrospectively reviewed. This was an exploratory sample of convenience and the duration was based on the availability of the operator (MN). Sex, complete course of antenatal corticosteroids, gestational age at birth, birth weight, birth weight *z*-score (9), postnatal age at the time of ultrasound, invasive ventilation, administration of postnatal steroids or non-depolarising muscle relaxing agents were recorded. As the echocardiograms were performed for clinical reasons, the study was registered with the Clinical Governance Department of King's College Hospital NHS Foundation Trust. The Health Research Authority Toolkit of the National Health System, UK confirmed that the study was not considered as research and hence would not need regulatory approval by a research ethics committee.

### Patent ductus arteriosus

Ventilated infants with a requirement for supplemental oxygen exceeding 40%, underwent echocardiographic assessment and the ones with a PDA were treated with ibuprofen or paracetamol before attempting extubation. Volume targeted ventilation was the mode of ventilation with a targeted tidal volume of 5–6 ml/kg and a positive end expiratory pressure of 5 cm H_2_O. The echocardiograms of the infants were reviewed by one author (MN), a neonatologist trained in echocardiography and further reviewed by a paediatric cardiologist (AB). Normal cardiac anatomy was confirmed in all included infants and the ductus was classified as “PDA” if a haemodynamically significant PDA was present, or “no PDA” if no haemodynamically significant PDA was detected. Defining the haemodynamic significance was based on the PDA shunt volume and its impact on the systemic and pulmonary circulation and myocardial function evaluation of the increased preload (10). The echocardiograms were performed for clinical indications (suitability for extubation or presence of murmur) and the diaphragmatic assessment was concurrently performed as part of the same scan.

### Diaphragmatic assessment

The right hemidiaphragm was assessed from the right subcostal area in the right lateral sagittal imaging plane in the midclavicular line (2). A 12–4 MHz linear probe was used with the direction of the ultrasound probe perpendicular to the diaphragm. From the M-mode trace, the mean inspiratory velocity of the diaphragm was calculated as:

$$V_{I}=Dx/t_{i}$$

**Figure 1:**
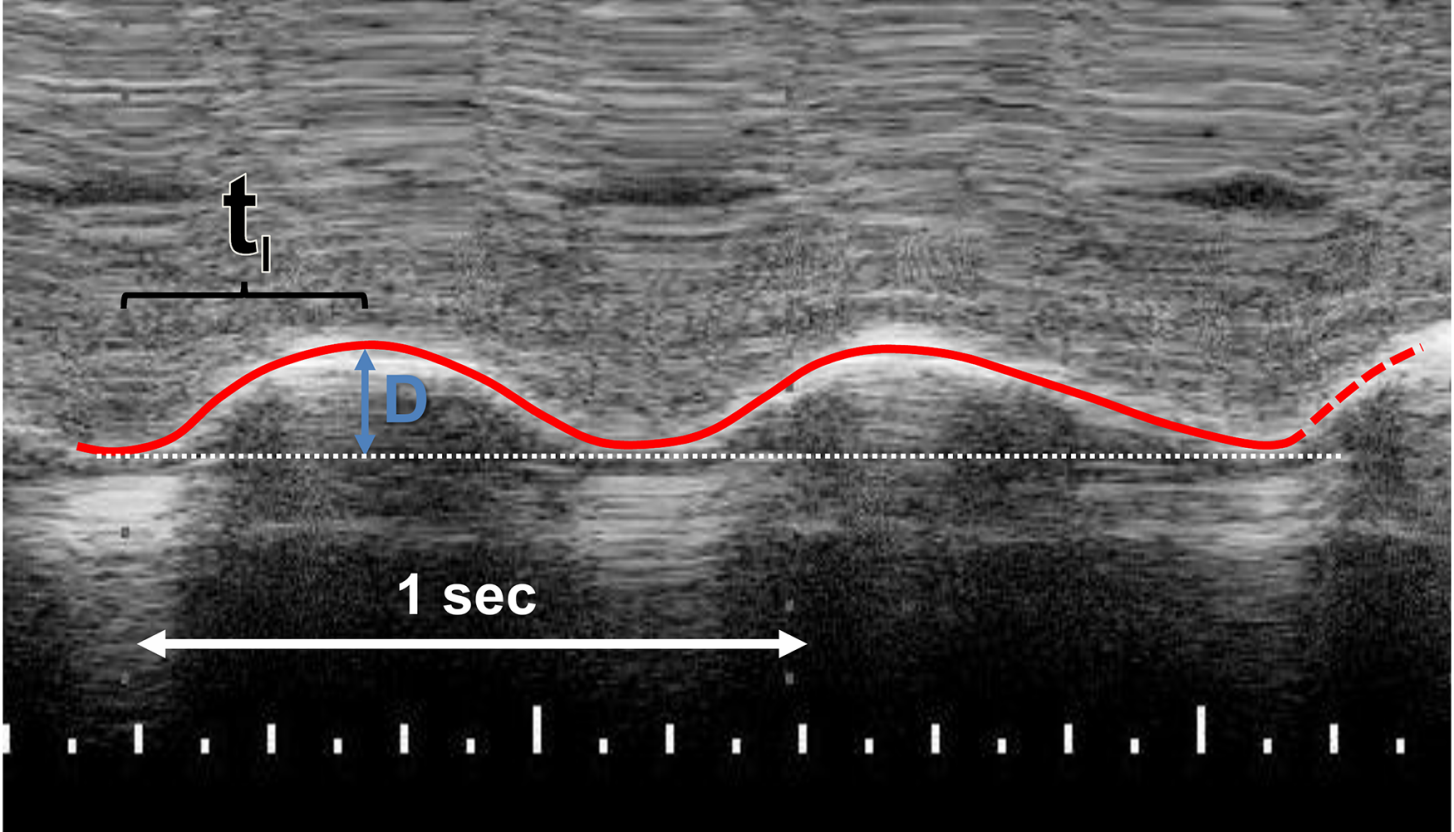
M-mode recording of the right posterior hemidiaphragm in right lateral sagittal plane. The mean inspiratory velocity was calculated by dividing the distance (**D**) that the right hemidiaphragm moves during inspiration by the inspiratory time (*T_I_*).

Where *D* was the distance the right posterior hemidiaphragm moved during inspiration divided by the inspiratory time (*t_I_*) (Figure 1) (5). The diaphragmatic thickness in the zone of apposition was also measured. The mean value of three to five repeatable measurements was calculated. The diaphragmatic ultrasound assessments were all performed by the same operator (MN).

### Statistical analysis

Data were tested for normality with the Kolmogorov–Smirnov test and found to be non-normally distributed. Continuous data were presented as median and interquartile range (IQR). Differences in diaphragmatic ultrasonographic parameters between infants with PDA and infants without PDA were tested for significance using the Mann–Whitney rank sum test. Differences in sex, antenatal steroids, gestational age, birth weight *z*-score and age at study were tested using the Mann–Whitney rank sum test for continuous variables or chi square test for binary variables. Multivariable linear regression analysis with the enter method and the *V_I_* as the outcome variable was used to adjust for differences in gestational age in the *V_I_* between infants with a PDA and infants without a PDA. The gestational age was included in the model as a potential confounder as it was significantly different (*p* < 0.05) between infants with a PDA and infants without a PDA. Variables without normal distribution were logarithmically transformed. Multi-collinearity among the independent variables in the multiple regression analysis was assessed by calculation of the tolerance for the independent variables. Linear regression analysis was used to graphically present the relationship of the *V_I_* with gestational age according to PDA status. The power of the study was calculated post-hoc based on the observed values of the *V_I_* in the groups of infants with and without a PDA.

Statistical analysis was performed using IBM SPSS Software (IBM, Chicago, IL).

## Results

During the study period, 17 echocardiographs with diaphragmatic assessment were undertaken in 14 infants (10 male) with a median (IQR) gestational age of 26.1 (25.8–30.6) [range: 23.7–39.0] weeks, birth weight of 780 (660–1385) gr, birth weight *z*-score of 0.43 (−1.59 to 1.20) and studied at a postnatal age of 18 (14–34) days. None of the included infants had been exposed to postnatal dexamethasone or non-depolarising muscle relaxing agents before the study.

Eight echocardiograms had evidence of a significant PDA and the remaining nine did not. The demographic and clinical characteristics of the included infants according to PDA status are presented in Table 1. The median (IQR) *V_I_* was significantly lower in scans with PDA [1.01 (0.78–1.86) cm/s] compared to scans without PDA [3.21 (2.80–3.59) cm/s, *p* < 0.001]. The median (IQR) D was significantly lower in scans with PDA [0.47 (0.40–0.77) cm] compared to scans without PDA [0.93 (0.67–1.39) cm, *p* = 0.021]. The median (IQR) *t_I_* was significantly longer in scans with a PDA [0.47 (0.36–0.56) s] compared to scans without a PDA [0.29 (0.22–0.43) s, *p* = 0.021]. The median (IQR) diaphragmatic thickness was not significantly different in scans with a PDA [4.0 (3.5–5.3) mm] compared to scans without a PDA [3.7 (2.7–5.5) mm, *p* = 0.574].

**Table 1:** Demographic and clinical characteristics of the study cohort. Median (IQR) or *N* (%).

	PDA	No PDA
	*N *= 8 scans	*N *= 9 scans
Male sex	6 (75)	4 (44)
Antenatal steroids	3 (38) (64)	6 (67)
Birth weight (gr)	740 (655–870)	1,185 (665–2910)
Birth weight *z* score	0.78 (−1.12 to 0.83)	0.01 (−1.86 to 1.79)
Gestational age (weeks)	25.8 (25.6–27.3)	29.0 (26.1–35.1)
Age at study (days)	31 (17–39)	14 (12–23)
Ventilated at study	5 (63)	5 (56)

**Figure 2:**
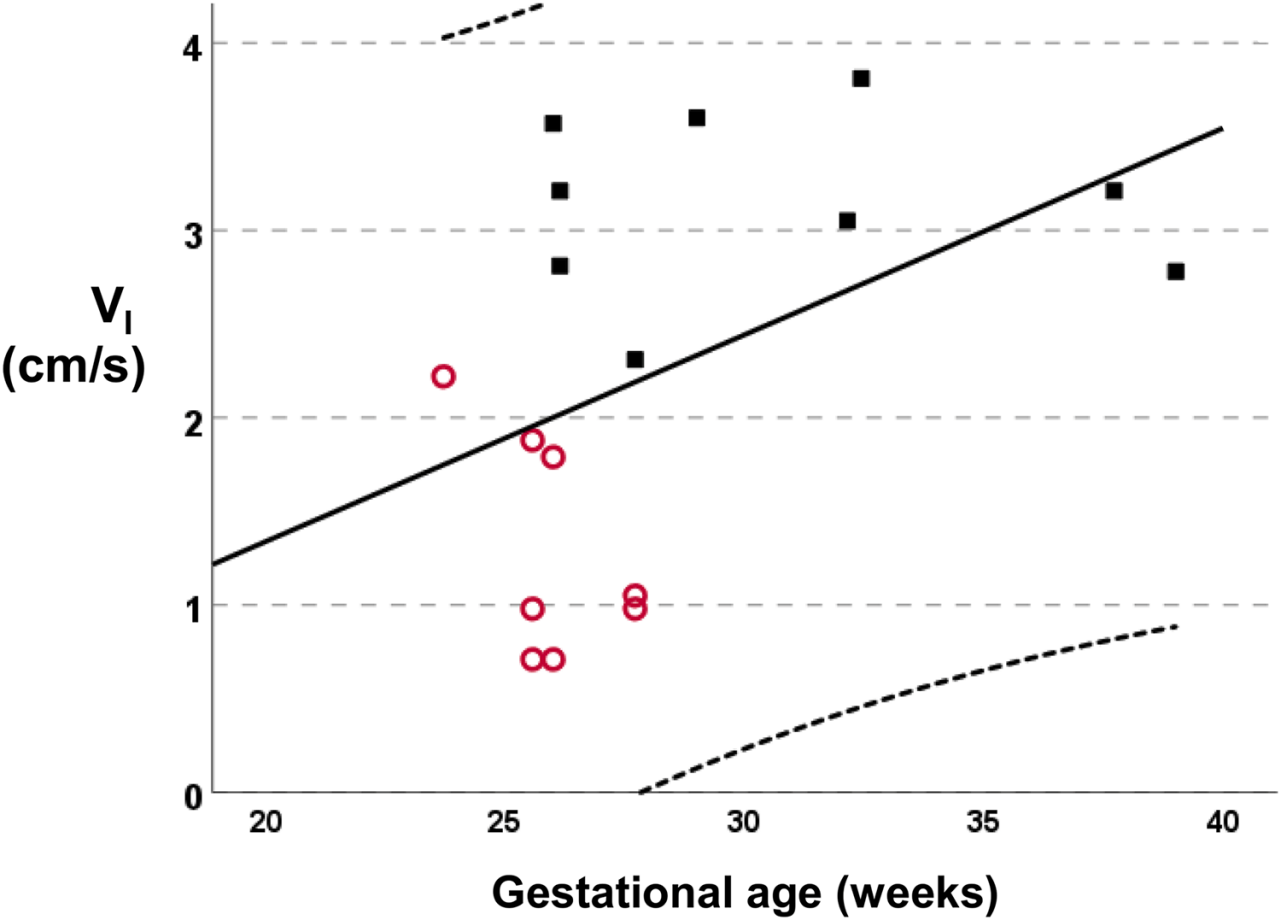
Linear regression analysis of the mean inspiratory velocity (*V_I_*) vs. the gestational age (*R*^2 ^= 0.197, standardised beta coefficient = 0.44, *p* = 0.074). Subjects with a PDA are depicted as empty circles and subjects without a PDA as solid squares.

The median (IQR) gestational age was lower in infants with a PDA [25.8 (25.6–27.3) weeks] compared to infants without a PDA [29.0 (26.1–35.1) weeks, *p* = 0.007]. The linear regression analysis of the gestational age with the *V_I_* is presented in Figure 2 (*R*^2 ^= 0.197, standardised beta coefficient = 0.44, *p* = 0.074). The median (IQR) birth weight was not different in infants with a PDA [740 (655–879) gr] compared to infants without a PDA [1,185 (660–2910) gr, *p* = 0.321]. The incidence of PDA in male infants (5 of 11) was not significantly different compared to the incidence of PDA in female infants (0 of 3, *p* = 0.145). The incidence of PDA in infants that were exposed to antenatal steroids (3 of 9) was not significantly different compared to the incidence of PDA in infants that were not exposed to antenatal steroids (2 of 5, *p* = 0.803). The incidence of PDA in scans done in invasively ventilated infants (6 of 10) was not significantly different compared to the incidence of PDA in scans of infants that were not invasively ventilated (2 of 7, *p* = 0.608). Seven assessments were made in infants that were on non-invasive support: three assessments were done on high flow nasal cannulae, three on continuous positive airway pressure and one while the infant was self-ventilating unassisted in room air. Following multivariable linear regression analysis, *V_I_* was independently associated with a PDA (adjusted *p* < 0.001, unstandardised beta coefficient: −1.94, 95% Confidence Interval = −1.26 to −2.62) but not with the gestational age (adjusted *p* = 0.659, unstandardised beta coefficient: −0.017, 95% Confidence Interval = −0.97 to 0.06).

Two infants had more than one echocardiogram and diaphragmatic ultrasound. The first infant had two scans and was diagnosed with PDA in both scans. The *V_I_* for the two scans was 0.71 and 0.98 cm/s on day 28 and 56 respectively. The second infant had three scans: he did not have a PDA in the first assessment on day 14 of life and the *V_I_* was 3.57 cm/s, and had a PDA in the next two consecutive scans on 34 and 40 day of life. In both consecutive scans with a PDA the *V_I_* was lower at 0.71 and 1.79 cm/s.

For the number of the included infants with and without a PDA and the observed difference in the *V_I_* between the two groups, the power of the study was calculated post-hoc at 96.9%, for a significance level of 0.05.

## Discussion

We have demonstrated that infants with a patent ductus arteriosus exhibit a lower mean inspiratory velocity compared to infants without a patent ductus arteriosus. This effect was independent of gestational age or other possible confounders.

To our knowledge no previous study has investigated the effect of a haemodynamically significant PDA on diaphragmatic kinetics in infants. Adult and animal studies have reported the possible mechanisms *via* which left ventricular heart failure (as encountered in significant PDA) could affect diaphragmatic function. Such mechanisms include hypo-perfusion and ischemia of the diaphragm (8), increased proteolytic activity and increased activation of proinflammatory cytokines such as interleukin 6 (IL6) and tumor necrosis factor–*α* (TNF-α) (11–13). Indeed, adult patients with heart failure exhibited lower maximum inspiratory pressures compared to controls (14) and impaired contractility of the diaphragm measured using the “twitch” transdiaphragmatic pressure (15). A study of a minipig animal model of heart failure demonstrated a shift in diaphragmatic fibers from type IIa (fast twitch type—resistant to fatigue) to type I (slow twitch type—prone to fatigue) (16) and increased levels of circulating pro-inflammatory IL-6 and TNF-α have been shown to directly impair muscle function in animal models (17, 18). The pathophysiological connection of impaired diaphragmatic kinetics and haemodynamically significant PDA is a novel finding in the newborn population. This observation might partially explain the inability of some infants to sustain independent breathing and why higher rates of PDA are seen in premature infants that fail extubation compared to the ones that successfully wean off invasive support (7).

In our study we used diaphragmatic kinetics *via* M-mode ultrasonography to assess diaphragmatic function. Other methods can be alternatively utilized, which may be considered methodologically superior, such as the measurement of maximum inspiratory pressures, the tension time index of the diaphragm or twitch transdiaphragmatic pressures (19). These methods however are methodologically more complex and require expensive specialised equipment which is not routinely available on neonatal units. Ultrasound, however, is readily available, inexpensive and familiar to neonatal clinicians as they use it regularly for assessing other systems such as the heart and brain. Furthermore, methods such as the maximum inspiratory pressures describe the respiratory muscles, only in relation to their capacity to generate strength and cannot describe properties of endurance or resistance to fatigue (19). Diaphragmatic kinetics, on the contrary, and the measurement of the mean inspiratory velocity have been shown to decrease significantly post induced muscle fatigue (5). We should note that although there is strong pathophysiological evidence in adults and neonates that diaphragmatic fatigue would manifest with impaired kinetics and reduced inspiratory velocity (10, 20), the mean inspiratory velocity *per se* is not a validated measure of respiratory muscle function in neonatal care nor are there reference values of the mean inspiratory velocity in newborn infants. Our study has highlighted the potential utility of this index but further and larger studies in different populations should be performed, including analyses of intra and inter-observer correlation, before this index could be clinically applied in everyday neonatal care.

In our study diaphragmatic thickness was not different in infants with PDA compared to the ones without PDA. Previous studies have reported that the diaphragmatic thickness was higher in preterm infants that were successfully extubated from invasive support compared to infants who failed to wean off invasive support (21) and lower in infants with bronchopulmonary dysplasia compared with healthy, age-matched controls (22). This discrepancy might be explained by population and methodological differences.

We should acknowledge as a limitation the limited size of our cohort and the observative nature of our study. There was, however, a clear separation of the values of the mean inspiratory velocity in infants with a PDA compared to the ones without a PDA, which implies that our sample was sufficient to elucidate this key difference. We could not infer from our study whether PDA was causative of impaired diaphragmatic kinetics as it was an observation cohort study. The next line of research might thus be a randomised study where infants with PDA are assigned to extubation after either diaphragmatic assessment or clinical decision alone, and evaluate the predictive ability of ultrasound to differentiate successful extubation. We did not assess in our study other factors that might influence respiratory muscle function in the newborn such as systemic or respiratory infection (23, 24) or respiratory mechanics (25). The diagnosis of infection, however, is often elusive in neonatal intensive care where unwell and premature infants with multiple comorbidities often receive broad spectrum antibiotics, while the incidence of true culture-positive sepsis is below one percent (26). Furthermore, respiratory mechanics would be distorted by hyperinflation in the chronic phase of the lung disease (25) and our population was studied earlier, at a median age of 18 days. Although in our cohort the gestational age in infants with PDA was lower compared to infants without PDA, the linear regression of gestational age and the *V*_*I*_ was not statistically significant (*p* = 0.074) probably due to a limited sample size. We have not included in our study results, the precise values of the level of invasive respiratory support but our preferred mode of ventilation was volume-targeted with a constant positive end expiratory pressure which would have mitigated excesses of over or under-ventilation.

In conclusion, we demonstrated that haemodynamically significant patent ductus arteriosus was associated with lower diaphragmatic inspiratory velocity and a possible negative effect on diaphragmatic performance This finding might partially explain the affected infants’ inability to successfully wean off invasive respiratory support.

## Data Availability

The raw data supporting the conclusions of this article will be made available by the authors, without undue reservation.
